# Efficacy of atomoxetine plus oxybutynin in the treatment of obstructive sleep apnea with moderate pharyngeal collapsibility

**DOI:** 10.1007/s11325-022-02634-x

**Published:** 2022-05-13

**Authors:** Paula K. Schweitzer, James P. Maynard, Paul E. Wylie, Helene A. Emsellem, Scott A. Sands

**Affiliations:** 1 Sleep Medicine and Research Center, St. Luke’s Hospital, 232 South Woods Mill Road, Chesterfield, MO 63017 USA; 2 CTI Clinical Trial and Consulting, Cincinnati, OH USA; 3 Arkansas Center of Sleep Medicine, Little Rock, AR USA; 4 The Center for Sleep & Wake Disorders, Chevy Chase, MD, and Department of Neurology (Clinical Faculty), George Washington University Medical Center, Washington, DC USA; 5 Division of Sleep and Circadian Disorders, Brigham and Women’s Hospital and Harvard Medical School, Boston, MA USA

**Keywords:** Obstructive sleep apnea, AD036, Atomoxetine, Oxybutynin, OSA pharmacotherapy

## Abstract

**Purpose:**

Preliminary studies have shown a significant decrease in severity of obstructive sleep apnea (OSA) with the use of a combination of atomoxetine and oxybutynin, with patients having moderate pharyngeal collapsibility during sleep more likely to respond. This study evaluated the efficacy and safety of AD036 (atomoxetine 80 mg and oxybutynin 5 mg) in the treatment of OSA.

**Methods:**

This trial was a phase 2, randomized, placebo-controlled crossover study comparing AD036, atomoxetine 80 mg alone, and placebo during three home sleep studies, each separated by about 1 week. The trial included patients with OSA and moderate pharyngeal collapsibility as defined by a higher proportion of hypopneas to apneas and mild oxygen desaturation.

**Results:**

Of 62 patients who were randomized, 60 were included in efficacy analyses. The apnea–hypopnea index (AHI) from a median (interquartile range) of 14.2 (5.4 to 22.3) events/h on placebo to 6.2 (2.8 to 13.6) with AD036 and 4.8 (1.4 to 11.6) with atomoxetine alone (*p* < .0001). Both drugs also decreased the oxygen desaturation index (ODI) and the hypoxic burden (*p* < .0001). AD036, but not atomoxetine alone, reduced the respiratory arousal index and improved ventilation at the respiratory arousal threshold (greater *V*_active_). There was a trend for total sleep time to be decreased more with atomoxetine alone than with AD036. The most common adverse event was insomnia (12% with AD036, 18% with atomoxetine).

**Conclusion:**

AD036 significantly improved OSA severity in patients with moderate pharyngeal collapsibility. Atomoxetine may account for the majority of improvement in OSA severity, while the addition of oxybutynin may mitigate the disruptive effect of atomoxetine on sleep and further improve ventilation.

**Trial registration:**

Clinical trial registered with www.clinicaltrials.gov (NCT04445688).

## Introduction

Obstructive sleep apnea (OSA) is a common sleep disorder, affecting over 930 million adults globally, and approximately 17% of women and 34% of men in the USA [1, 2]. Untreated OSA is associated with increased incidence of hypertension, coronary heart disease, arrhythmias, heart failure, and stroke [3]. Continuous positive airway pressure (CPAP), the primary therapy for OSA, is very effective, but its use is limited by poor tolerance and adherence in a substantial number of patients [4–6]. The principal alternative treatment options, which include mandibular advancement devices and upper airway surgery, are not effective in all patients, and prediction of efficacy is challenging [2]. There are currently no approved pharmacotherapies for OSA, and efforts to develop such therapies have been generally unsuccessful [7, 8]. However, recent advances in our understanding of the pathophysiological traits which contribute to OSA indicate that airway collapsibility and pharyngeal dilator muscle function are two of the most important traits contributing to OSA, suggesting that drugs that are capable of raising baseline upper airway muscle activity and its responsiveness to increased load have the potential to resolve OSA [9, 10]. Preliminary studies have shown a significant decrease in OSA severity using norepinephrine reuptake inhibitors such as atomoxetine and reboxetine in combination with antimuscarinics such as oxybutynin or hyoscine butylbromide [11–13]. In one study, the combined administration of atomoxetine and oxybutynin resulted in a reduction in the apnea–hypopnea index (AHI) by approximately 60% in a small group of patients with OSA [11]. The presumed mechanism for these findings is an increase in upper airway dilator muscle activity and responsiveness caused by noradrenergic and antimuscarinic stimulation [14, 15].

AD036 is a fixed-dose combination of atomoxetine and oxybutynin that is being developed for the treatment of OSA. Post hoc analyses of feasibility studies assessing several dose combinations of AD036 in patients with varying OSA severity and physiologic characteristics indicated that patients with clinical signs of moderate pharyngeal collapsibility during sleep (see below) were more likely to respond to the medication than those with more severe collapsibility [16]. This study was undertaken to assess the efficacy and safety of AD036 in this patient population with moderate pharyngeal collapsibility.

## Methods

### Study design

This trial was a phase 2, randomized, 3-period, single-dose, placebo-controlled crossover study to evaluate the efficacy and safety of AD036 versus placebo or atomoxetine alone in the treatment of OSA. The study was conducted at five clinical investigative sites in the USA and was approved by institutional review boards at each site and performed in accordance with the Declaration of Helsinki. All participants provided written informed consent (www.clinicaltrials.gov identifier NCT04445688).

### Participants

Participants were adults with OSA who had low to moderate pharyngeal collapsibility during sleep as indicated by respiratory criteria based on either a baseline polysomnography (PSG) study from a prior AD036 study or a home sleep apnea test (HSAT) during screening. Respiratory criteria for moderate collapsibility were derived from previous analysis collected in the academic [16, 17] and sponsored research settings (unpublished observation) and included (1) an AHI = 10 to < 20, or (2) AHI ≥ 20 along with one of the following criteria: mean oxygen desaturation of obstructive events ≤ 4%, fraction of hypopneas > 90%, or fraction of hypopneas 50–90% with mean oxygen desaturation 4–8%. Previous studies (unpublished) found that these criteria indicated moderate pharyngeal collapsibility using the methods of Sands and Wellman [18]. Hypopneas were defined using a minimum oxygen desaturation criterion of 4% [19]. Demographic requirements included age 25–65 years (up to 67 for participants of the prior AD036 study) and body mass index (BMI) between 18.5 and 40 kg/m^2^. Key exclusion criteria were as follows: clinically important craniofacial malformation, cardiac disease, neurological disorder, cognitive dysfunction, constipation, gastric retention, urinary retention; history of narcolepsy, schizophrenia, schizoaffective disorder, bipolar disorder, suicide attempt or ideation in the past year; substance use disorder (within past 2 years) or positive screen for drugs of abuse; use of oxygen or medications with central nervous system effects.

### Treatments and study protocol

After screening for eligibility, participants underwent three overnight HSATs after dosing of one of the following treatments on each study night: AD036 (comprised of commercially available formulations of atomoxetine 80 mg and oxybutynin 5 mg), atomoxetine 80 mg + placebo, placebo + placebo. Participants were centrally randomized to one of six treatment sequences in this three-period crossover design. There was no blinding, but the study treatments were packaged in a manner to reduce participants’ knowledge of the treatment assignment. A 1-week minimum washout period occurred between each of the crossover nights. Participants were instructed to take study drug orally immediately prior to bedtime. HSATs were utilized instead of in-lab PSGs to decrease the risk of transmission of coronavirus disease 2019 (COVID-19) as the study was initiated at the beginning of the COVID-19 pandemic. HSATs were conducted utilizing the Nox A1 system (Nox Medical, Reykjavık, Iceland) and included electroencephalogram (EEG), electrooculogram, respiratory airflow (using nasal pressure transducer), thoracic and abdominal respiratory effort, oximetry, heart rate, and body position. Because scalp EEG and submental electromyogram (EMG) leads are difficult to self-apply, we used only frontal leads for EEG recordings and excluded submental EMG leads. Site personnel contacted participants via video call during each HSAT night to assist with sensor and equipment placement, and to confirm study drug dosing. Adverse event and concomitant medication monitoring were conducted on HSAT nights and during each washout period. CPAP use was not permitted within 2 weeks of the first HSAT or anytime thereafter. Other treatments for OSA, including oral, nasal, and positional devices, were not permitted during study participation.

### Data analysis

Sleep studies were scored by a blinded central reading center (Sleep Strategies Inc., Ontario, Canada) using American Academy of Sleep Medicine guidelines [19]. The 4% oxygen desaturation criterion was used for scoring of hypopneas and subsequent calculation of the AHI, and for calculation of the oxygen desaturation index (ODI). We also calculated AHI using the 3% desaturation or arousal criterion for hypopneas (AHI_3A_). Sleep staging was scored using the frontal EEG leads. In addition to standard sleep and respiratory variables, we also calculated hypoxic burden, a measure of the severity of oxygen desaturation over the entire night determined by quantifying the respiratory event–associated area under the desaturation curve from a pre-event baseline. This metric has been shown to predict adverse cardiovascular outcomes better than AHI [20]. We also calculated the fraction of hypopneas to total disordered breathing events (apneas plus hypopneas), arousals associated with respiratory events, and arousal burden [21], a validated measure which quantifies both the frequency and intensity of respiratory-related arousals expressed as the sum of arousal intensities per hour of sleep (arousal intensity ranging from 0 to 9). Finally, a validated algorithm was used to estimate endotypic traits, including passive airway collapsibility (*V*_min_; ventilation measured during obstructive events at the minimal ventilatory drive), pharyngeal muscle activation (*V*_active_; ventilation measured at maximum ventilatory drive during obstructive events prior to arousal), loop gain (stability of the respiratory control system), and arousal threshold (estimated ventilatory drive prior to arousal) [18].

### Statistical analysis

The primary efficacy endpoint was the comparison of AHI between AD036 and placebo. Secondary endpoints were comparisons between AD036 and placebo and between AD036 and atomoxetine alone on hypoxic burden and ODI. Tertiary endpoints included total time with oxygen saturation (SaO_2_) < 90%, sleep stage distribution, and respiratory arousal index.

Approximately 54 participants (9 per sequence) were planned for enrollment. This sample size provided 90% power to detect a treatment difference in AHI of 11 events/h at a two-sided 0.05 significance level, using a within-subject standard deviation of 12 events/h.

Continuous variables were analyzed using a within-subject analysis of variance (ANOVA) model including treatment sequence, treatment, and visit as fixed effects. Robust variance estimates for the fixed effects were used for testing treatment differences. Within-subject variability was modeled using an unstructured covariance pattern. Endotypic traits and arousal burden were analyzed post hoc using a mixed model analysis with treatment as a fixed factor and subjects as a random factor. Data analyses were performed using SAS for Windows, version 9.4 or higher (SAS, Cary, NC). Data are presented as median and interquartile range or least square (LS) mean with 95% confidence interval.

Baseline (study entry criteria) data were not used in analyses because the time between the baseline sleep study and the initiation of this protocol was quiet variable (between 1 and 18 months earlier), and the type of study conducted was a full PSG for some and an HSAT for others. Thus, the placebo arm of the trial was used as the comparator for the medication arms.

## Results

### Participants

**Fig. 1:**
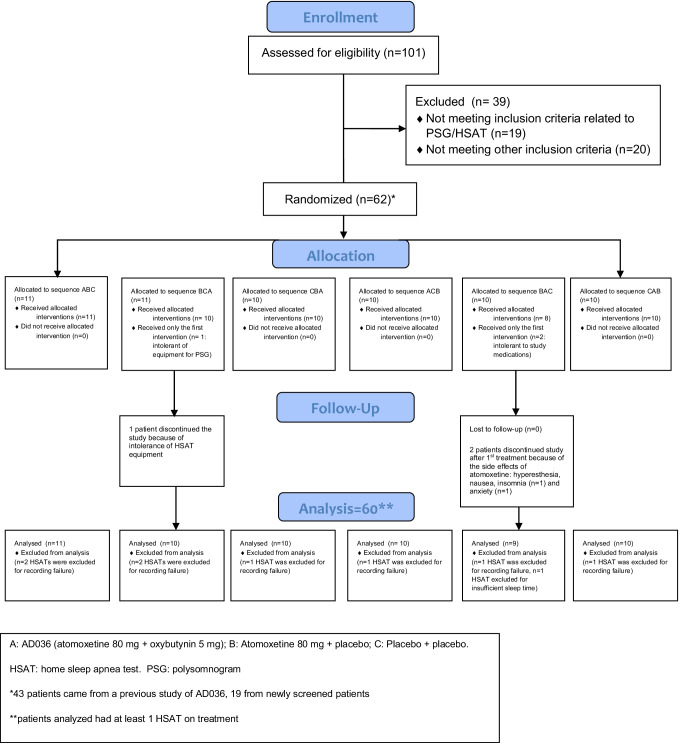
Consolidated Standards of Reporting Trials diagram of the clinical trial

A total of 62 participants were randomized and 59 (95%) completed all three treatments (Fig. 1). Three participants discontinued study participation following the first study night (two because of treatment-emergent adverse events, one because of discomfort with the HSAT device). Safety analysis was conducted using all 62 participants (safety population). Efficacy analyses were conducted using the modified intent-to-treat population, which included all participants who took at least 1 dose of any study treatment and had at least one measurement on the primary endpoint (*n* = 60).

Participants in the safety population were 50% male, primarily white (66%), and non-Hispanic (94%). Median age was 53.0 years (range: 28 to 66) and median BMI was 32.3 kg/m^2^ (range: 22.6 to 39.6).

### Efficacy

**Fig. 2:**
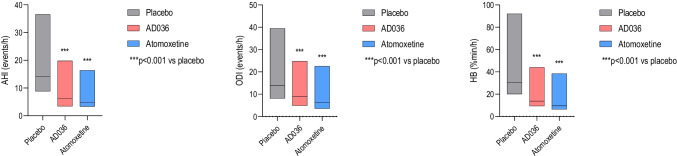
Box plots of apnea–hypopnea index (AHI), oxygen desaturation index (ODI), and hypoxic burden (HB) on placebo, AD036, and atomoxetine. Black lines indicate medians; boxes indicate 25th and 75th percentiles

Efficacy data are presented in Table 1. AD036 was superior to placebo for the primary and secondary endpoints of AHI, ODI, and hypoxic burden (Fig. 2). AD036 also resulted in a decrease in time spent with SaO2 < 90% and an increase in the fraction of hypopneas comprising the AHI vs. placebo. The respiratory arousal index was lower for AD036 compared to both placebo and atomoxetine. However, there was no difference between AD036 and atomoxetine in most other respiratory measures, with atomoxetine alone yielding a significant reduction in AHI, ODI, and hypoxic burden when compared to placebo. Post hoc analysis of arousal burden showed a decrease with AD036 compared to both placebo and atomoxetine (Table 2).

**Table 1:** Obstructive sleep apnea variables and sleep architecture during placebo, AD036, and atomoxetine conditions

	Placebo		AD036		Atomoxetine	
	Median (IQR)	LS mean (95% CI)	Median (IQR)	LS mean (95% CI)	Median (IQR)	LS mean (95% CI)
AHI, events/h	14.2 (5.4 to 22.3)	18.8 (14.5 to 23.1)	6.2 (2.8 to 13.6)	11.5*** (7.2 to 15.8)	4.8 (1.4 to 11.6)	11.5*** (7.2 to 15.8)
NREM AHI, events/h	11.1 (3.6 to 20.3)	16.7 (12.4 to 21.0)	5.7 (2.6 to 12.8)	11.9** (7.5 to 16.3)	4.4 (1.5 to 12.6)	11.3** (6.9 to 15.6)
REM AHI^1^, events/h	26.4 (9.1 to 46.1)	30.9 (24.2 to 37.6)	10.2 (3.1 to 24.8)	16.4* (6.9 to 25.9)	12.8 (0 to 30.7)	15.9* (7.2 to 24.6)
AHI_3A_, events/h	23.6 (12.4 to 32.7)	27.1 (22.3 to 31.9)	14.0 (8.1 to 26.2)	20.1*** (15.3 to 24.9)	15.4 (9.0 to 27.9)	21.6*** (16.8 to 26.4)
ODI, events/h	13.9 (5.8 to 25.6)	20.1 (15.8 to 24.4)	9.0 (4.1 to 15.8)	12.8*** (8.4 to 17.1)	6.4 (2.8 to 16.2)	13.3*** (9.0 to 17.7)
Hypoxic burden, % min/h	30.5 (10.4 to 61.6)	46.1 (35.4 to 56.8)	13.7 (4.4 to 30.3)	24.3*** (13.5 to 35.1)	9.7 (3.3 to 28.8)	26.8*** (16.1 to 37.6)
SaO_2_ < 90%, min	14.1 (2.7 to 49.8)	40.3 (27.9 to 52.6)	5.1 (0.6 to 29)	22.2*** (9.7 to 34.8)	2.2 (0.3 to 20.9)	16.9*** (4.5 to 29.2)
Respiratory arousal index, events/h	9.0 (5.5 to 13.9)	13.0 (9.6 to 16.5)	7.0 (3.1 to 13)	10.4*# (6.9 to 13.9)	7.5 (4.3 to 16.4)	13.4 (10.0 to 16.9)
Non-respiratory arousal index, events/h	10.3 (7.2 to 13.6)	11.2 (8.9 to 13.5)	15.8 (9.7 to 24.6)	18.3*** (15.9 to 20.6)	17.4 (10.2 to 25.3)	18.8*** (16.4 to 21.1)
Fraction of hypopneas, % AHI	59.4 (39.0 to 76.8)	71.9 (67.3 to 76.5)	79.1 (61.9 to 94.2)	88.6*** (83.9 to 93.2)	88.9 (64.7 to 98.3)	89.8*** (85.1 to 94.4)
Total sleep time, min	376.5 (324.5 to 417.3)	364.7 (343.8 to 385.5)	347.5 (255.6 to 404.3)	329.7* (302.0 to 357.5)	315.5 (255.0 to 363.8)	303.1*** (280.5 to 325.7)
Sleep efficiency, % time in bed	86.3 (79.8 to 90.8)	83.5 (80.5 to 86.4)	76.5 (61.7 to 86.5)	72.9*** (67.8 to 78.0)	72.7 (58.6 to 82.4)	70.1*** (66.0 to 74.3)
N1, %TST	10.3 (6.5 to 17.8)	13.4 (11.1 to 15.8)	12.8 (7.7 to 17.5)	14.3 (12.0 to 16.6)	13.7 (7.8 to 21.1)	15.3 (13.0 to 17.7)
N2, %TST	62.1 (49.1 to 68.9)	59.9 (56.3 to 63.5)	60.3 (47.6 to 69.5)	58.5 (54.4 to 62.6)	59.0 (46.0 to 69.7)	56.1 (51.6 to 60.6)
N3, %TST	0.8 (0 to 4.9)	3.0 (1.6 to 4.5)	0.2 (0 to 2.9)	2.1 (1.1 to 3.2)	0.2 (0 to 3.0)	2.0* (1.1 to 2.9)
REM, %TST	11.5 (3.7 to 20.0)	11.2 (8.8 to 13.6)	0.0 (0 to 6.3)	3.2*** (1.8 to 4.6)	0.0 (0 to 0.7)	1.7*** (0.4 to 3.0)

^*^p < .05 vs. placebo, **p < .01 vs. placebo, ***p < .0001 vs. placebo, #*p* < .05 vs. atomoxetine

^1^REM AHI was calculated only if REM time was greater than 5 min and TST was greater than 120 min (*N* = 47 for placebo, 17 for AD036, 12 for atomoxetine)

*95%CI*, 95% confidence interval; *AHI*, apnea hypopnea index using 4% desaturation criterion for hypopneas; *AHI*_*3A*_, apnea hypopnea index using 3% desaturation or arousal criterion for hypopneas; *IQR*, interquartile range; *LS*, least squares; *N1*, stage 1; *N2*, stage 2; *N3*, stage 3; *ODI*, oxygen desaturation index; *REM*, rapid eye movement; *SaO*_*2*_, oxygen saturation; *TST*, total sleep time

**Table 2:** Results of mixed effect model testing of AD036 and atomoxetine on endotypic traits and arousal burden

	Collapsibility (*V*_min_, % eupnea)	Pharyngeal muscle activation (*V*_active_, % eupnea)	Arousal threshold (% eupnea)	Loop gain (unitless)	Arousal burden (intensity units/h)
Placebo	45 [40 to 49]	88 [79 to 97]	130 [120 to 140]	0.58 [0.54 to 0.63]	50 [36 to 65]
AD036 (change from placebo)	7 [3 to 11] *P* < 0.001	9 [1 to 18] *p* = 0.03	− 15 [− 24 to − 6] *p* = 0.001	− 0.07 [− 0.11 to − 0.03] *p* < 0.001	− 12 [− 23 to − 1] *p* = 0.029
Atomoxetine 80 mg (change from placebo)	8 [4 to 11] *p* < 0.001	6 [− 1.5 to 14] *p* = 0.11	− 16 [− 25 to − 7] *p* < 0.001	− 0.08 [− 0.12 to − 0.04] *p* < 0.001	2 [− 9 to 12] *p* = 0.787

Results are reported as mean [95% confidence interval]. *V*_min_ describes the ventilation observed at minimal drive (lowest decile) during obstructive events. *V*_active_ represents the ventilation measured at maximum ventilatory drive during obstructive events. Loop gain describes the stability of the respiratory control system. Arousal threshold is calculated as the estimated ventilatory drive prior to arousal. Values for *V*_min_ and *V*_active_ do not represent observed data but rather the underlying collapsibility derived from a sigmoidal transformation function to handle the ceiling effects previously described for this type of data [17]. Arousal burden represents the product of arousal intensity and the arousal frequency and was calculated by the summation of all respiratory-related arousal intensities during sleep divided by the total sleep duration [20]; the intensity of each arousal is represented by a 0–9-point scale where 9 is the most intense arousal

Total sleep time was decreased by both drugs, with median reduction compared to placebo of 29.0 min for AD036 and 61.0 min for atomoxetine (Table 1). There was a trend for lower sleep time on atomoxetine vs. AD036 (*p* = 0.06). Both drugs reduced REM sleep.

### Effects on endotypic traits

The post hoc analyses of endotypic traits are presented in Table 2. Compared to placebo, both AD036 and atomoxetine improved passive airway collapsibility (*V*_min_), reduced the arousal threshold, and reduced loop gain. AD036, but not atomoxetine, increased *V*_active_, compared to placebo.

### Safety

No serious adverse events were reported during the study. A total of 78 treatment-emergent adverse events were reported in 26 (42%) participants in the safety population: 21 (36%) individuals with AD036; 18 (29%) with atomoxetine; and 5 (9%) with placebo. Two participants discontinued study participation because of adverse events considered related to study treatment with atomoxetine (anxiety in one person, hyperesthesia, nausea, and insomnia in one person.) A third participant discontinued participation because of discomfort with the HSAT device on the atomoxetine-treatment night. The most common adverse events for AD036 were insomnia (12%) and nausea (5%); for atomoxetine, insomnia (18%), nausea (5%), decreased appetite (5%), and feeling jittery (5%); and, for placebo, insomnia (7%). All adverse events were rated as mild to moderate except for one event (hyperesthesia) that was rated as severe.

## Discussion

The main finding of this study is that AD036 significantly improved AHI and other measures of OSA severity during a single night of treatment. The magnitude of effect was clinically meaningful, with a 54% reduction in median AHI compared to placebo. On AD036 vs. placebo, residual respiratory events were milder, as indicated by an increase in the fraction of hypopneas comprising the AHI (from a median of 73% on placebo to 94% with AD036) and substantial reduction in the time spent with SaO2 < 90% (from a median 14.1 min to 5.1 min), as well as a decrease in hypoxic burden (from a median 30.5% min/h to 13.7% min/h). However, the effects of AD036 on AHI and most other respiratory endpoints were not significantly different from that of atomoxetine alone. A noted exception was a reduction in respiratory arousals with AD036 but not with atomoxetine alone, an effect seen with the respiratory arousal index (22% decrease) as well as with arousal burden (24% decrease), a related measure that quantifies both intensity and frequency of respiratory arousals. In addition, the post hoc analysis of endotypic traits indicated that only AD036 increased *V*_active_, suggesting that AD036 had a stronger effect than atomoxetine alone in improving ventilation by recruiting the upper airway dilator muscles, consistent with prior findings [11, 16]. AD036 was also less disruptive of sleep than atomoxetine alone, with a median decrease in total sleep time compared to placebo of 29.0 min for AD036 versus 61.0 min for atomoxetine alone.

Both AD036 and atomoxetine alone were safe and well tolerated in this single-night crossover exposure. The most common adverse events (insomnia and nausea with AD036; insomnia, nausea, decreased appetite, jitteriness with atomoxetine) were consistent with the expected profile of the individual approved drugs, atomoxetine and oxybutynin [22, 23]. Insomnia, the most common side effect, was reported more frequently with atomoxetine alone (18%) than with AD036 (12%), consistent with the objective sleep data*.*

These data extend the findings of a previous pilot study demonstrating improvement in AHI and other respiratory measures with the combination of atomoxetine and oxybutynin [11]. Unlike that study, we also found improvement in AHI with atomoxetine alone, with no difference between atomoxetine alone and the combination of atomoxetine-oxybutynin for most respiratory measures. Our data also differs from a study of atomoxetine 40–80 mg alone that showed no reduction in AHI or respiratory disturbance index after 4 weeks of use [24]. Differences in results may possibly be related to study design, sample size, or the specific focus on patients with milder collapsibility in the current study. The study by Sangal et al. [24] included 15 patients with very mild sleep apnea in a pre-post study design without placebo control. The evaluation of atomoxetine alone in the Taranto-Montemurro et al. study [11] occurred in a supplementary open-label investigation in a subsample (*n* = 9) of participants from the primary study and also found no effect of atomoxetine on AHI, although it was responsible for 38% improvement in ventilation during sleep, compared to the 18% improvement with oxybutynin alone [16]. Furthermore, in the previous trial, atomoxetine alone reduced the arousal threshold (patients woke up more easily due to obstructive events) and reduced loop gain (breathing control was more stable).

Results of endotypic trait analyses were similar to those of the prior atomoxetine-oxybutynin study [16]. Both AD036 and atomoxetine alone improved airway collapsibility (greater *V*_min_) and improved breathing stability (reduced loop gain), but only AD036 improved ventilation at the respiratory arousal threshold (greater *V*_active_), suggesting a stronger effect on upper airway dilator muscles. Both AD036 and atomoxetine alone reduced the arousal threshold by about 15%, an effect that may decrease sleep efficiency and increase the likelihood of arousal to small changes in ventilatory drive. The reduction in the arousal threshold may be a consequence of the adrenergic effect of atomoxetine or the result of spontaneous arousals during mild flow limitation and may be a limitation to its use.

One limitation of the current study was the use of a single night for each treatment condition. This is a potential problem as severity of OSA can vary from night to night, particularly in individuals with mild to moderate OSA [25, 26]. Our study population, a sample chosen to have moderate pharyngeal collapsibility, was comprised of individuals with mostly mild to moderate OSA as defined by AHI. Furthermore, 12 participants who met AHI entry criteria based on a prior study had AHI < 5 on the placebo night.

We do not believe that data quality issues with the use of HSATs in lieu of in-lab PSGs affected the study results. However, a validated [27], non-standard recording montage was used to facilitate the self-application of sensors by the patients. The use of this alternative montage may have affected the quantification of sleep staging, especially REM and N3, which were notably low even on the placebo night. REM was absent in 18%, 56%, and 70% of participants on the placebo, AD036, and atomoxetine nights, respectively. However, prior studies of atomoxetine [24] and atomoxetine combined with oxybutynin [16, 28] in patients with sleep apnea have shown reduced or absent REM while using standard in-lab PSG. It is unlikely that the reduction in REM sleep in both drug conditions in the current study accounts for the majority of decrease in the overall AHI, since sensitivity analysis of the AHI specifically during NREM sleep confirmed the main findings.

In conclusion, the current study demonstrated that AD036 improves AHI and other measures of OSA severity in a population of patients with moderate pharyngeal collapsibility as described by a higher proportion of hypopneas to apnea and mild degree of oxygen desaturation. The data suggest that atomoxetine is primarily responsible for the improvement in respiratory variables, while the addition of oxybutynin ameliorates the disruptive effect of atomoxetine on sleep and further improves ventilation. Longer-duration studies on a broader group of patients with more-diverse ethnicities are needed to determine if these findings persist over time and to further describe the characteristics of patients likely to respond to this type of pharmacologic treatment for OSA. The use of standard PSG recordings and quality-of-life metrics will be helpful to further elucidate the impact of these drug combinations on sleep architecture and daytime functioning.

## Data Availability

The datasets generated during and/or analyzed during the current study are available from the sponsor at ltaranto@apnimed.com on reasonable request.
